# Australians’ Well-Being and Resilience During COVID-19: The Role of Trust, Misinformation, Intolerance of Uncertainty, and Locus of Control

**DOI:** 10.3390/jcm13247495

**Published:** 2024-12-10

**Authors:** Nida Denson, Kevin M. Dunn, Alanna Kamp, Jehonathan Ben, Daniel Pitman, Rachel Sharples, Grace Lim, Yin Paradies, Craig McGarty

**Affiliations:** 1 School of Social Sciences, Western Sydney University, Penrith, NSW 2751, Australia; a.kamp@westernsydney.edu.au (A.K.); j.ben@westernsydney.edu.au (J.B.); d.pitman@westernsydney.edu.au (D.P.); r.sharples@westernsydney.edu.au (R.S.); grace.lim@outlook.com.au (G.L.); 2 Office of the Deputy Vice-Chancellor (Research, Enterprise and International), Western Sydney University, Penrith, NSW 2751, Australia; k.dunn@westernsydney.edu.au; 3 Alfred Deakin Institute for Citizenship and Globalisation, Deakin University, Geelong, VIC 3220, Australia; yin.paradies@deakin.edu.au; 4 School of Psychology, Western Sydney University, Penrith, NSW 2751, Australia; c.mcgarty@westernsydney.edu.au

**Keywords:** COVID-19 pandemic, psychological factors, mental health, loneliness, resilience, Australia

## Abstract

**Background/Objectives**: In response to the COVID-19 pandemic, Australian state and federal governments enacted boarder closures, social distancing measures, and lockdowns. By the end of October 2020, the 112-day lockdown in the Australian state of Victoria was the longest continuous lockdown period internationally. Previous studies have examined how the COVID-19 pandemic and government restrictions have affected Australians’ mental health and well-being; however, less is known about the relationship between psychological variables and well-being. **Methods**: We administered a national survey of Australians aged 16 years and over (*N* = 1380) in November 2020 to examine the psychological factors that promoted and hindered Australians’ well-being and resilience during the first year of the pandemic. **Results**: Our study found that Australians reported normal to moderate levels of anxiety, moderate stress, mild depression, and moderate to high loneliness. Interpersonal trust was consistently a protective factor for well-being and resilience and was associated with less depression, anxiety, stress, and loneliness, and greater resilience. Participants with greater inhibitory anxiety (intolerance of uncertainty) and an external locus of control were more likely to be depressed, anxious, stressed, and lonely, and less resilient, compared with those with less inhibitory anxiety and those who believed that these outcomes were determined by their own actions. COVID-19 beliefs were associated with more depression, anxiety, stress, and resilience. **Conclusions**: This study seeks to inform the development of mental-health, well-being, and resilience strategies by government agencies, non-government organisations, and healthcare providers in times of crisis and in “ordinary” times.

## 1. Introduction

The COVID-19 pandemic created a global physical and mental health crisis, with far-reaching economic, social, political, and ethical consequences that remain felt today, more than four years after its outbreak [1,2]. Populations across the globe have faced unprecedented levels of socioeconomic uncertainty while needing to respond to the virus by adopting and adapting to preventative health measures, restrictions on daily proximities and activities, and fragmented societal structures [3]. Taking place across diverse contexts (e.g., geopolitically, socio-culturally), the pandemic affected countries differently, and some of its effects have been acutely uneven [2,4,5]. National, state, and local responses to the pandemic have varied, including the use of preventative measures [6], reigniting long-standing concerns about readiness and systemic inequality in health responses, both within and between countries [7,8].

By November 2020, Australia had 27,893 confirmed cases of COVID-19 and 907 deaths [9]. Compared with other countries, Australia’s relatively low cases were attributed to fairly successful compliance with border closures and social distancing measures to prevent the virus’s spread [10]. Key amongst such measures were several “lockdowns” (i.e., stay-at-home orders) to minimise outbreaks. Such measures had immediate impacts on the employment and financial security of individuals. When the current study was conducted, the unemployment rate in Australia was 6.8% [11], an increase of 1.7% from before the pandemic in 2019 [12], and 22% of businesses reported revenue decreases [13].

International research has demonstrated that the COVID-19 government restrictions contributed to adverse mental health and well-being outcomes, such as increased loneliness, depression, anxiety, and financial worry and reduced social support [14,15,16]. Similar effects were found in the United States and United Kingdom, revealing that the pandemic increased loneliness and poor mental health amongst adults [17,18]. A US study found that stay-at-home orders were associated with increased anxiety and financial worry as well as increased loneliness [19]. Research on previous pandemics has also found negative impacts on mental health outcomes, for example, increased levels of anxiety during the 2009 H1N1 pandemic [20] and the 2003 SARS outbreak [21]. However, the fuller, long-term extent of the COVID-19 pandemic’s (and its associated transformations’) negative mental health implications is still unclear, and researchers continue to grapple with issues that range from “long-COVID” to persisting racial disparities in pandemic health outcomes [4].

Numerous Australian studies have examined the well-being and resilience of Australians during the COVID-19 pandemic. Most notable is the ANU Social Research Centre’s COVID-19 Impact Monitoring Series [22]. They administered 11 surveys over the first 2 years of the pandemic. They found that life satisfaction decreased substantially at the beginning (during the first few months of the pandemic) and then fluctuated after that. In terms of psychological distress, there was a large increase in in the beginning, and again, it fluctuated after that. Social interaction has seen a dramatic decrease, which in 2022 had not yet returned to pre-pandemic levels, and has contributed to increased social isolation and loneliness. They also found that social cohesion (trust, fairness, helpfulness) increased significantly in the first six months of the pandemic and has slightly decreased since, but remains above pre-COVID levels [22]. Notably, at the beginning of the pandemic, they found that Australians had a high level of trust and confidence in the government and systems [22]. Another study on Australian adolescents found that 53% experienced worsening physical health, and 75% experienced worsening mental health as a result of the pandemic [23]. Unfortunately, these negative impacts on physical and mental health were exacerbated for adolescents with a history of depression or anxiety. There were also negative impacts of the pandemic on Australian adolescents’ interpersonal relationships, with 66% reporting feeling less connected to their friends, and 68% reported that the pandemic had a negative impact on family stress.

These studies have focused on various factors that have impacted the well-being and resilience of Australians during the initial impacts of COVID-19, such as social isolation and economic uncertainty. However, one aspect that has been relatively understudied is the relationship of psychological variables on mental health and well-being. We were particularly interested in the potential influence of trust (trust in government and interpersonal trust), belief in COVID-19 misinformation, intolerance of uncertainty, and locus of control. These variables have been shown to be important factors in pandemics, shaping how societies mitigate the impact of COVID-19. Trust in government (or political trust) is the extent to which people “trust in political actors and institutions”, and interpersonal trust (or social trust) refers to “trust in other people” [24], with a recent meta-analysis showing that the object of trust (e.g., government, president) moderates the relationship between trust and various pandemic behaviours such as vaccine uptake and compliance with public health measures [24].

As has been shown in other times of uncertainty, another influencing factor that emerged during the pandemic was an increase in beliefs in conspiracy theories (i.e., explanations for the causes of events or circumstances secretly plotted by multiple powerful actors) [25] and misinformation (inaccurate or false information) [26,27]. At the time this study was conducted (November 2020), there were various beliefs, including beliefs in conspiracy theories, circulating on social media, such as that COVID-19 was a bio-engineered virus created by the Chinese, COVID-19 is worsened through installing 5G towers, and hot temperatures kill the virus [28]. Belief in conspiracy theories, especially as they relate to COVID-19, has been shown to be associated with a decrease in institutional trust (trust towards the police, state authorities, politicians, experts, and hospitals/doctors), less support of governmental regulations to curb the virus (e.g., closing schools), and lower likelihood of adopting physical distancing measures (e.g., limiting physical contact) [29]. Belief in conspiracy theories has also been shown to be associated with perceptions of lower risk to one’s own health and the health of others, but higher perceptions of risk to the economy and human liberties, which in turn were both associated with lower compliance with governmental COVID-19 guidelines [30]. Understanding beliefs in conspiracy theories will help elucidate both the public health challenges they pose as well as broader patterns of how societies respond to large-scale threats, making them an important focus of any comprehensive analysis of pandemic response and behaviour.

Personality characteristics, such as intolerance of uncertainty (the inability to tolerate uncertainty and ambiguous situations) and having an external locus of control (beliefs that whatever happens to them is beyond their control) have also been shown to affect how people responded to the COVID-19 pandemic, with both being associated with increased mental health outcomes such as depression, anxiety, and stress [31,32]. The current study sought to provide this evidence by gauging the extent to which Australians’ mental health and well-being have been affected by the COVID-19 pandemic and the psychological variables that promoted and hindered their well-being and resilience in the context of the pandemic. This study was part of a larger project on enhancing resilience and social belonging during the pandemic, and a subset of the findings has been presented in the project’s final report [33]. The current study included the investigation of the following research questions:

(1) During the first year of the pandemic, how did Australians fare in terms of their well-being and resilience?
(2) What were the psychological factors that promoted or hindered Australians’ well-being and resilience in the first year of the COVID-19 pandemic?

Our second research question has the following associated hypotheses:

**Hypothesis** **1 (H1).** *Participants who have higher trust in government will report better mental health, less loneliness, and higher resilience.*

**Hypothesis** **2 (H2).** *Participants who have higher interpersonal trust will report better mental health, less loneliness, and higher resilience.*

**Hypothesis** **3 (H3).** *Participants who hold various COVID-19 beliefs (including conspiracy theories and misinformation) will report poorer mental health, more loneliness, and less resilience.*

**Hypothesis** **4 (H4).** *Participants who are more intolerant of uncertainty will report poorer mental health, more loneliness, and less resilience.*

**Hypothesis** **5 (H5).** *Participants who have an external locus of control will report poorer mental health, more loneliness, and less resilience.*

## 2. Materials and Methods

### 2.1. Participants and Procedure

This study received ethics approval from the Western Sydney University Human Research Ethics Committee (H14042 on 27 October 2020). We administered a national online survey to Australians between 12 and 26 November 2020. Inclusion criteria consisted of Australian residents 16+ years of age. We included 16- and 17-year-olds, as they have the “maturity and capacity” to make an “informed decision” [34]. Even the Australian Psychological Society’s (APS) ethical guidelines deem that minors 14–15 years of age have sufficient maturity to give voluntary informed consent to participate in research [34]. One empirical study examined the capacity of 4th, 7th, and 10th graders and university students to understand their research rights. While understanding generally increased with age, this study found that 10th graders’ (ranging from 14.9–17.6 years of age) understanding of the purpose and nature of the research, research risks and benefits, confidentiality, and voluntary nature of participation, and the right to withdraw, did not differ from adults’ understanding [35]. In particular, 4th and 7th graders did not fully understand the right to withdraw. Given the low-risk nature of the survey, it was appropriate to include 16–17-year-olds in this study [35].

We utilised Dynata, an online panel provider, which has a pool of over 300,000 Australian panellists (including 16–17-year-olds). Dynata (https://www.dynata.com/about/ (accessed on 13 September 2024)) is one of the world’s largest first-party data platforms and survey service companies. With a global reach, Dynata has a strong Australian presence with a diverse panel, and it can also accommodate multiple data collection methods (e.g., online surveys, mobile surveys, telephone interviews). Our inclusion criteria consisted of Australian residents 16 years of age and older. Our exclusion criteria are non-Australian residents as well as Australian residents aged 15 years and younger. We targeted a national sample that was representative by state/territory, with a 1:2 ratio of those from culturally and linguistically diverse (CALD) backgrounds to those from Anglo backgrounds in order to obtain a culturally diverse sample. Dynata contacts the panellists, providing information about the research and asking them if they would like to participate in the study. They are also informed that their participation is entirely voluntary and that their responses will be anonymous. Only interested participants proceeded to completing the survey. The final sample consisted of 1380 respondents who were currently residing in Australia and were 16 years of age and older.

Our sample was representative by state/territory [36] and income [37]. In terms of geographic distribution, 31.0% were in New South Wales, 25.0% in Victoria, 21.6% in Queensland, 11.0% in Western Australia, 7.3% in South Australia, 1.9% in Tasmania, 1.5% in the Australian Capital Territory, and 0.7% in the Northern Territory. Almost half (48.9%) of the participants earned <AUD 40,000 per annum, and 42.6% self-ranked themselves to be in the lower half in Australia in subjective social status. Almost three-fifths (57.1%) identified as female (compared with 51% nationally [38]). A significant number of participants were born overseas (39.4% compared with 29% nationally [38]), and the majority had an Anglo background (76.5%), including many Anglo migrants inadvertently. Our sample was highly educated, with almost half with at least a tertiary level qualification (45.0%), including 14.0% with a postgraduate qualification, compared with 26.3% and 6.5% at the national level, respectively [39]. Only 1.5% of participants were 16–17 years of age, with older participants being over-represented (i.e., 60.5% of participants were aged 55+ compared with 29.6% nationally [40]). Unemployment was somewhat high (7.7%), slightly higher than the national average of 6.8% at the time [11]. In terms of political affiliation, the distribution was fairly representative of the current Australian political climate, with 33.6% most likely to vote for the Labour party and 41.1% most likely to vote for the Liberal-National Coalition. Almost four-fifths (78.4%) lived in a major city, 16.6% lived in an inner regional area, with the remaining 5.0% in an outer regional, remote, or very remote area. 

### 2.2. Survey

The survey comprised questions with closed-ended response options (quantitative) on demographic and background characteristics, trust/distrust in government, interpersonal trust, belief in COVID-19 misinformation, individual differences variables (e.g., intolerance of uncertainty, locus of control), mental health (e.g., depression, anxiety, stress), loneliness, and resilience (see Table S1).

#### 2.2.1. Outcome Variables

Depression/Anxiety/Stress [41]: We utilised the DASS-9 to represent depression, anxiety, and stress (9 items, α = 0.932). The scale consists of nine items grouped into three factors—depression (e.g., “I felt that I had nothing to look forward to”), anxiety (e.g., “I felt I was close to panic”), and stress (e.g., “I found myself getting agitated”). Respondents rated their feelings of depression, anxiety, and stress over the past week (from 1 = did not apply to me at all to 4 = applied to me very much or most of the time—almost always). The severity of general psychological distress was indicated by adding all DASS items and was interpreted as normal (0–6), mild (7–8), moderate (9–10), severe (11–12), and extremely severe (≥13).

Loneliness [42]: Loneliness was assessed using the University of California Los Angeles Loneliness Scale (8 items; α = 0.870). Respondents rated their perceived social isolation (from 1 = I often feel this way to 4 = I never feel this way), which was reverse coded so that higher scores indicated greater loneliness.

Resilience [43]: Resilience was assessed using the Brief Resilience Scale (6 items; α = 0.893) and asked respondents to rate their ability to bounce back and recover from stress (from 1 = strongly disagree to 5 = strongly agree), with higher scores indicating greater resilience.

#### 2.2.2. Predictor Variables

Trust in federal and state government during the pandemic: Trust in federal government (5 items; α = 0.914) was a newly established scale (e.g., “The federal government is providing me with sufficient information about who should be isolated in response to COVID-19”). Trust in state government (4 items; α = 0.914) was also a newly established scale (e.g., “My state government is providing me with sufficient information about who should be isolated in response to COVID-19”). Respondents rated their agreement (from 1 = strongly disagree to 7 = strongly agree). 

Distrust of government in general: Distrust of government was adapted from the Political Cynicism Scale [44] (5 items; α = 0.811). Sample items include “The government is regulated by a few big interests” and “The government wastes considerable tax money” (from 1 = strongly disagree to 7 = strongly agree).

Interpersonal trust [45]: Interpersonal trust included items from the Trusting Scale from the International Personality Item Pool (https://ipip.ori.org/ (accessed on 13 September 2024)). The scale consisted of 6 items (α = 0.834), and respondents were asked to rate the accuracy of each statement (e.g., “I believe in human goodness”) from 1 = very inaccurate to 5 = very accurate.

COVID-19 beliefs (including conspiracy theories and misinformation): This was a newly developed scale of COVID-19 beliefs that included both conspiracy theories as well as misinformation. Conspiracy theories are explanations for causes of events or circumstances secretly plotted by powerful actors, and misinformation is inaccurate or false information. The items for this scale were based on various news articles circulating at the time, and included 10 items (α = 0.892). Sample items include “COVID-19 is a bio-engineered virus” and “COVID-19 is worsened through installing 5G towers” (from 1 = strongly disagree to 7 = strongly agree).

Intolerance of uncertainty (IOU) [46]: IOU was assessed using the Intolerance of Uncertainty Scale, which is “the tendency of an individual to consider the possibility of a negative event occurring to be unacceptable, irrespective of the probability of occurrence”. The IOU scale has two subscales: inhibitory anxiety (5 items; α = 0.889) and prospective anxiety (7 items; α = 0.836). Inhibitory anxiety refers to uncertainty inhibiting action or experience (e.g., “The smallest doubt can stop me from acting”), while prospective anxiety refers to fear and anxiety based on future events (e.g., “I always want to know what the future has in store for me”). This scale asks respondents to rate their ability to tolerate uncertainty and ambiguous situations (from 1 = not at all characteristic of me to 5 = entirely characteristic of me). Higher scores indicate greater intolerance of uncertainty.

External locus of control (LOC) [47]: External LOC was assessed using the Locus of Control of Behaviour Scale (17 items; α = 0.841). The scale asks respondents to score items relating to their belief about control over their personal behaviour (from 1 = strongly disagree to 6 = strongly agree). Internality items were transposed prior to summing scores, with higher scores indicating externality (e.g., “A great deal of what happens to me is probably just a matter of chance”).

#### 2.2.3. Control Variables

We controlled for a number of variables in our analyses which have been shown to have a relationship with mental health and well-being in previous research [22]. Gender was a dichotomous variable (1 = female; 2 = male). Age was an ordinal variable (from 1 = 16–17 years of age to 8 = 75 years and over). Country of birth was coded as a dichotomous variable (1 = Australia; 2 = not Australia), as was Anglo background (1 = Anglo background; 2 = non-Anglo background). Both education (from 1 = no formal qualifications to 6 = postgraduate qualification) and income (from 1 = under AUD 20,000 to 6 = AUD 150,000 or more) were ordinal variables. Subjective social status was adapted from the MacArthur Scale of Subjective Social Status [48]. Participants were shown a ladder representing people in Australia and were asked to indicate where on the hierarchy they perceived their current standing relative to other people in Australia (from 1 = “bottom” to 10 = “top”). Employment was a series of dummy variables (i.e., employed, unemployed, self-employed, caring/home duties, student) with people who are retired as the reference/comparison group. Living arrangements was also a series of dummy variables (i.e., single person living alone, single person living with parents/family, single person living with children, couple living with children, living in a share house with unrelated adults) with couples without children as the reference/comparison group. Remoteness was an ordinal variable (1 = major cities, 2 = inner regional, 3 = outer regional/remote/very remote), and having one or more disabilities was dichotomous (1 = no, 2 = yes). Lastly, having been negatively impacted by the 2019–2020 bushfires in Australia was an ordinal variable (1 = not at all to 5 = extremely so). In 2019–2020, Australia experienced the most catastrophic bushfire season on record, burning over 24 million hectares and over 3000 homes, alongside almost 3 billion animals displaced, harmed, or killed [49].

#### 2.2.4. Statistical Methods

We conducted both descriptive (i.e., means, frequencies) and inferential statistical analyses (i.e., bivariate correlations, standard multiple regressions) to provide a snapshot of Australians’ well-being and resilience in the first year of the pandemic, as well as the correlates between psychological factors and well-being and resilience during COVID-19. Bivariate correlations measure the strength and direction of the relationship (or association) between two variables. Bivariate correlations range between −1.0 and +1.0. A correlation of zero and non-significant correlations indicate that there is no linear relationship between two variables. A significant positive correlation indicates that an increase in one variable is associated with an increase in another variable, and a significant negative correlation indicates that an increase in one variable is associated with a decrease in another variable. The bivariate correlations were followed up with a series of standard multiple regression analyses. Multiple regression can be considered an extension of correlations. While bivariate correlations can compare only two variables, multiple regression can examine how multiple independent variables predict a single dependent variable. One advantage of multiple regression is that we can assess the extent to which an independent variable is related to a dependent variable while simultaneously controlling for a number of independent variables. All analyses were conducted using SPSS (Version 29).

## 3. Results

### 3.1. Well-Being and Resilience

**Figure 1:**
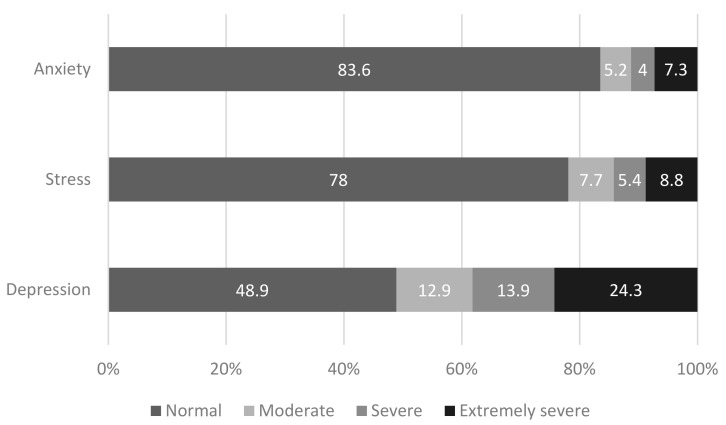
Anxiety, stress, and depression during the COVID-19 pandemic (*N* = 1380).

In regard to mental health, the respondents reported “normal to moderate” anxiety (M = 1.51, SD = 2.07), “moderate” stress (M = 2.15, SD = 2.11), and “mild” depression (M = 2.18, SD = 2.30) (Table 1). In terms of frequencies, 16.4% of respondents were in the “moderate to extremely severe” range for anxiety, 22% were in the “moderate to extremely severe” range for stress, and 51.1% were in the “moderate to extremely severe” range for depression (Figure 1). More than three-fifths (63.8%) of respondents reported “moderate to high” loneliness (M = 22.77, SD = 5.79) (Table 1). And 28.6% reported “low” resilience (M = 3.31, SD = 0.83) (Table 1). 

### 3.2. Psychological Factors, Well-Being, and Resilience

Table 2 presents the bivariate correlations across all the variables. The two psychological variables that had the strongest correlations with the outcomes were an external locus of control (Hypothesis 5) and inhibitory anxiety (Hypothesis 4). In particular, having an external locus of control (e.g., “A great deal of what happens to me is probably just a matter of chance”) was consistently associated with mental health, loneliness, and resilience (Hypothesis 5). Specifically, those who believed that luck or randomness determine their life’s outcomes were more likely to be depressed, anxious, stressed, lonely, and less resilient compared with those with an internal locus of control (i.e., those who believe that they control their own success). Similarly, those high in inhibitory anxiety (i.e., freeze in times of uncertainty) were also more likely to be more depressed, anxious, stressed, lonely, and less resilient than those who were lower on inhibitory anxiety (Hypothesis 4).

The variables that were moderately correlated with the outcomes were interpersonal trust (Hypothesis 2), prospective anxiety (Hypothesis 4), and COVID-19 beliefs (including conspiracy theories and misinformation) (Hypothesis 3). Those who trusted others were less depressed, anxious, stressed, and lonely, and were also more resilient (Hypothesis 2). This means that those who trust others in general tended to have better well-being and resilience. In addition, those who had higher levels of prospective anxiety (anxiety in anticipation of future uncertainty) had poorer mental health and were more lonely and less resilient than those who were lower in prospective anxiety (Hypothesis 4). And those who endorsed COVID-19 conspiracy theories and misinformation were more depressed, anxious, stressed, and lonely, and less resilient, compared with those who did not endorse these COVID-19 beliefs (Hypothesis 3). 

The smallest correlates with well-being and resilience were (dis)trust of government (Hypothesis 1). In particular, trust in the federal and state governments’ management of the pandemic was associated with better mental health, decreased loneliness, and higher resilience (Hypothesis 1). In a similar vein, being generally distrustful of government was associated with poorer mental health, increased loneliness, and less resilience compared with those who are generally trustful of government (Hypothesis 1). 

Table 3 presents the standardised beta coefficients for the standard multiple regression results when all the correlates (predictors) of well-being and resilience were considered simultaneously. Once socioeconomic background and demographic characteristics were taken into account, the strongest (and most consistent) predictors of poorer well-being and less resilience were having an external locus of control (Hypothesis 5) and greater inhibitory anxiety (Hypothesis 4). Both factors predicted greater depression, anxiety, stress and loneliness, and less resilience. Belief in COVID-19 misinformation was associated with poorer mental health (Hypothesis 3). In terms of resilience, people who had a general distrust of government were less resilient compared with those who generally trust the government (Hypothesis 1). In terms of protective factors, interpersonal trust predicted less depression, anxiety, stress, loneliness, and greater resilience (Hypothesis 2). Surprisingly, belief in COVID-19 misinformation was associated with greater resilience.

## 4. Discussion

Even though Australia was relatively successful in managing the COVID-19 pandemic, the pandemic and associated responses to the pandemic have had detrimental effects on the well-being and resilience of Australians [50,51,52,53]. This study contributes to research on the COVID-19 pandemic by examining the factors that were associated with promoting or hindering well-being and resilience during the first year of the pandemic. Our study highlights three important findings. 

First, the strongest negative correlates of well-being and resilience were having an external locus of control and high intolerance of uncertainty (specifically, inhibitory anxiety, which is uncertainty inhibiting action or experience). This finding is consistent with research that shows the importance of considering psychological factors affecting people’s well-being and resilience [54], and it points to the moderating effects of both these factors on the relationship between COVID-19 anxiety and quality of life [55]. Given all the uncertainty surrounding the COVID-19 pandemic (e.g., fear of contracting COVID-19, quarantine measures, extended travel restrictions), it makes sense that intolerance of uncertainty would be an influential factor in people’s reactions to the pandemic. A recent study even showed the mediating role of intolerance of uncertainty, and specifically, how it can amplify the negative effects of fear of the COVID-19 virus on depression, anxiety, and stress [56]. Our study extends the research in this area by disaggregating the subscales of intolerance of uncertainty, and in doing so, we demonstrate that inhibitory anxiety (rather than prospective anxiety) was the more influential factor associated with well-being and resilience (prospective anxiety had no association in the final analyses once we controlled for all other factors). Similarly, having an external locus of control (what happens to me is beyond my control) was also strongly associated with all the well-being and resilience outcomes in our study. This finding is consistent with other research, which has also shown that having an external locus of control is associated with increased depression, anxiety, and stress in the context of the COVID-19 pandemic [57]. Another study found that having an external locus of control exacerbated the relationship between psychological stress due to COVID-19 and depression and anxiety [58]. These two psychological variables had the strongest association with well-being and resilience out of all the variables in our study. 

Second, trust was also an important factor in shaping people’s well-being and resilience in the COVID-19 pandemic context. In this study, we examined both interpersonal trust as well as dis/trust in government. We found that interpersonal trust was a consistent protective factor for well-being and resilience. Those with higher interpersonal trust were less depressed, anxious, stressed, and lonely, and were more resilient, compared with those who were lower in interpersonal trust. This finding adds to the literature on the benefits of interpersonal trust on mental health and well-being in the pandemic context, with international research showing that manipulating interpersonal trust increases conscious compliance with pandemic prevention norms, such as reducing unnecessary outdoor activities, bringing an earlier end to virus spreading [59]. While trust in the federal and state governments’ responses to COVID-19 had no impact on any of the mental health and well-being outcomes, those more likely to distrust the government (generally speaking) showed lower resilience. Beyond the pandemic context, these findings align with research showing greater distrust is associated with poorer mental health [60] and that trust is central to well-being, with personal well-being being higher in societies with higher levels of institutional trust and interpersonal cooperation [61,62,63]. 

Our findings on the association between trust and well-being and resilience is also an important contribution to broader trust research and support calls to enhance and build trust. It is well established that “Trust is essential for the successful functioning of society—politically, economically, and socially” (p. 85, [64]). Trust in institutions and social trust is key to civic and social cooperation, which in turn propagates economic growth and institutional performance [64,65,66,67,68]. Prior to the pandemic, Australia was ranked one of the most “distrusting” nations [69]. During the pandemic, Australia moved from being a “distrusting” nation that sits around the global average to a “trusting” nation well above the global average [70]. However, in 2022, the Edelman survey [71] reported that Australia’s “trust bubble” had burst (p. 8), with Australia amongst countries showing an annual decline in trust in government, business, NGOs, and media (p. 5). While global trust continues to decline post-pandemic, Australia has once again improved its levels of trust, moving from one of the most distrusting nations in 2023 to “neutral” in 2024—having neutral trust in business, neutral trust in NGOs, neutral trust in government, and trust in employers, but continued distrust in the media. As argued by some, “these trends indicate that the levels of trust are dynamic, and that they can be improved and worsened depending on political settings and structural circumstances” (p.87, [63]). Given the associations between trust and well-being, the fluctuating levels of trust in Australia and around the world, and the flow-on effects of distrust on the successful functioning of society, we join others in arguing that raising levels of trust is imperative.

The third and last key finding is that COVID-19 beliefs (including conspiracy theories and misinformation) were associated with poorer mental health and greater resilience. On the one hand, those who believed in COVID-19 conspiracy beliefs and misinformation were more likely to be depressed, anxious, and stressed. On the other hand, these beliefs made them feel more resilient. While counterintuitive, this finding is consistent with Douglas’s theory on the psychology of conspiracy theories, as beliefs in conspiracy theories increase in times of crisis such as the COVID-19 pandemic, indicating that people’s psychological needs are not being met [72,73]. People are drawn to conspiracy theories since they satisfy their epistemic motives (the need to understand) as well as their existential motives (the need to feel safe and secure) [72]. Consistent with Douglas’s theory [72], we found that people who believe in COVID-19 conspiracy theories were more anxious, stressed, and depressed. However, believing in these conspiracy theories also made them feel more resilient in the sense of being able to “bounce back” after hard times, which is what our measure of resilience assessed. 

Our study has some important implications for policymakers and practitioners working to improve Australians’ mental health and resilience following the pandemic. First, the rates of depression and stress in Australia during the first year of the pandemic are comparable with the UK’s and higher than New Zealand’s, while the rates for anxiety were higher than both countries [74]. Uncertainty during COVID-19 may have been particularly rife in Australia and manifested as inhibitory anxiety with a significant impact on all well-being and resilience outcomes. While the lockdowns and stay-at-home orders in Australia may have been highly effective in preventing contagion, they would have had an especially deleterious effect on those with higher levels of inhibitory anxiety, which would have curtailed their personal interactions and exacerbated loneliness. Similarly, those who believed that their lives’ outcomes were determined by external forces and less under their own control had significantly poorer well-being and resilience. Beyond governmental organisations dedicated to improving mental health and services in Australia (e.g., the Australian Institute of Health and Welfare), key organisations working to address COVID-19 anxiety may want to further consider these findings in their service provision (e.g., Beyond Blue, Black Dog Institute, the Australian Psychological Society (APS)) and target people who have an external locus of control and who are high in inhibitory anxiety, as these people struggled the most during the pandemic. 

Second, social support systems are imperative to building resilience during a pandemic. In our study, we found that interpersonal trust was a consistent protective factor during the first year of the pandemic and improved the well-being and resilience of the respondents. A recent study on Australians’ trust found a positive and significant (although weak) association between age and social/interpersonal trust, such that as age increased, so too did the extent of these forms of trust [63]. These findings support the contact theory, which suggests that interpersonal contact between groups (which will expand over a person’s lifetime) can reduce prejudice [75] and lead to greater levels of trust between groups. To enhance resilience during difficult times, stakeholders should support systems, platforms, and practices that have protective roles, including the development and strengthening of close, meaningful connections and interpersonal contact and trust. Increased funding should be allocated to local, community-level organisations and initiatives that boost interpersonal trust and social connections. There has been an emerging agreement that lifting trust in government and key state agencies can be protective in crises, but our findings stress the importance of affirming interpersonal trust amongst citizens. Other work on trust [76] has asserted that while trust in government agencies is important, we also need to lift the trust we have in ordinary people. Government agencies need to trust the public, and this would have virtuous effects on interpersonal trust.

Third, our findings highlight the pervasiveness of beliefs in conspiracy theories and misinformation. This requires urgent attention due to the direct correlations between such beliefs and distrust in government [77,78,79], distrust in science [80], distrust of mainstream media [81,82,83], support for populist politics [84], and far-right extremism [81,85]. Adding to this anti-social context, our study has shown that belief in conspiracy theories and misinformation is associated with increased depression, anxiety, and stress. These findings support the recommendations by others to provide timely, accessible COVID-19 information and to contest misinformation as quickly as possible [72]. This is imperative, as conspiracy theories will thrive again in future pandemics and crises [73]. We echo the recommendations [86] for pandemic public health risk communication, particularly regarding the need for information that is clear, timely, balanced, and from a reputable source; for correcting mis/disinformation about the virus and its protective measures; and for promoting a sense of control and self-efficacy to help mitigate health threats. Indeed, “inoculating” people with factual information before they are exposed to conspiracy theories and mis/disinformation can curb the belief and spreading of conspiracy theories [72]. As a result, governments should support reliable information sources and news in order to provide accurate information as soon as possible in order to discourage any mis/disinformation that may follow. 

Another strategy is to promote collectivism, appealing to the larger group membership (e.g., “All of us Australians are in this together”) [72]. Previous research has demonstrated that individualists were more likely to believe in COVID-19 conspiracy theories and less likely to take preventative measures (e.g., getting the vaccine, wearing a mask); however, this was not the case for collectivists [87]. Thus, governments should promote collective messages, which can discourage beliefs in conspiracy theories, encourage preventative behaviours that are beneficial for all Australians, and mitigate any negative mental health outcomes associated with mis/disinformation and conspiracy theory beliefs. For example, since March 2020, New Zealand’s clear message to “Unite against COVID-19” has been displayed prominently on the government’s website as the country’s source of COVID-19 information (https://covid19.govt.nz/ (accessed on 13 September 2024)). The message received over 700 million views within the first 3 months [88]. Such campaigns and messaging must, however, go beyond broad calls for unity in the face of adversity and engage with disparities and inequities that have often characterised the pandemic, including its impacts and responses, while offering substantive and clear guidelines for action. Our findings on the importance of interpersonal trust affirm the utility of such a policy agenda.

### Limitations and Future Research

The current study had some limitations. First, the study used a correlational cross-sectional design, so we were unable to make any definitive causal claims about the factors that predict well-being and resilience outcomes. In other words, we cannot definitively conclude that belief in COVID-19 misinformation causes poorer mental health or vice versa. Longitudinal research could test our claims to examine whether these associations hold up in a longitudinal study that could definitely test causality. Second, our study was conducted in November 2020, during the first year of the pandemic, with most states implementing further lockdowns in 2021 [89,90,91]. Thus, our findings may not be generalizable past the first year of the pandemic, and other research could examine whether these relationships were sustained or if they changed in subsequent years of the pandemic. Third, our sample consisted only of participants aged 16 years and older, excluding young Australians 15 years and younger, so our findings cannot be generalised to younger cohorts. Future research should focus on young people in particular. Fourth, while the sample was representative in some aspects (i.e., state/territory, income), it was not completely representative of the Australian population at large; thus, future research is needed to examine the generalizability of this study’s findings. Lastly, we created a newly developed scale in relation to COVID-19 beliefs, given that there were no appropriate, already-established existing scales. The COVID-19 beliefs scale included conspiracy beliefs as well as misinformation. While it did have high internal reliability (α = 0.892), future research should examine the psychometric properties of this scale as well as the generalizability of this study’s findings.

## 5. Conclusions

Given that well-being and resilience may help individuals to cope more effectively with significant traumatic events such as COVID-19 [92], it is important that we continue to examine how we can increase Australians’ well-being and resilience so that we are better prepared for the next crisis or public health concern (e.g., Mpox [93]). In particular, increasing trust (interpersonal as well as trust in government) and providing people with timely factual information as soon as possible to curtail beliefs in misinformation are both promising avenues for future research. We hope the findings will also help inform the allocation of funding and resources towards improving the well-being and resilience of all Australians.

## Figures and Tables

**Table 1:** Descriptive statistics for the well-being and resilience variables.

Variable	Range	Mean	SD	# of Items	Alpha
Anxiety	0–9	1.51	2.07	3	0.853
Stress	0–9	2.15	2.11	3	0.837
Depression	0–9	2.18	2.30	3	0.867
Loneliness	8–32	22.77	5.79	8	0.870
Resilience	1–5	3.31	.83	6	0.889

**Table 2:** Correlations with well-being and resilience (*N* = 1380).

		1	2	3	4	5	6	7	8	9	10	11	*M*	*SD*
1. Trust in federal government	*r*	1	0.477	–0.447	0.186	−0.062	0.056	−0.052	−0.129	−0.105	−0.171	0.145	5.148	1.304
	*p*		<0.001	<0.001	<0.001	0.021	0.037	0.052	<0.001	<0.001	<0.001	<0.001		
2. Trust in state government	*r*		1	−0.243	0.171	−0.261	0.027	−0.041	−0.128	−0.072	−0.106	0.078	5.292	1.359
	*p*			<0.001	<0.001	<0.001	0.310	0.128	<0.001	0.008	<0.001	0.004		
3. Distrust of government in general	*r*			1	−0.333	0.221	0.171	0.195	0.195	0.211	0.207	−0.205	4.386	1.083
	*p*				<0.001	<0.001	<0.001	<0.001	<0.001	<0.001	<0.001	<0.001		
4. Interpersonal trust	*r*				1	−0.244	–0.310	−0.411	−0.440	−0.420	−0.433	0.430	3.443	0.743
	*p*					<0.001	<0.001	<0.001	<0.001	<0.001	<0.001	<0.001		
5. COVID-19 beliefs	*r*					1	0.191	0.297	0.320	0.343	0.153	−0.139	2.827	1.231
	*p*						<0.001	<0.001	<0.001	<0.001	<0.001	<0.001		
6. IOU: Prospective anxiety	*r*						1	0.715	0.369	0.466	0.306	−0.345	2.922	0.787
	*p*							<0.001	<0.001	<0.001	<0.001	<0.001		
7. IOU: Inhibitory anxiety	*r*							1	0.632	0.652	0.493	−0.557	2.407	0.980
	*p*								<0.001	<0.001	<0.001	<0.001		
8. External locus of control	*r*								1	0.627	0.519	−0.598	2.944	0.660
	*p*									<0.001	<0.001	<0.001		
9. Depression, anxiety, and stress ^a^	*r*									1	0.569	−0.553	5.806	5.912
	*p*										<0.001	<0.001		
10. Loneliness ^a^	*r*										1	−0.545	2.156	0.724
	*p*											<0.001		
11. Resilience ^b^	*r*											1	3.314	0.826
	*p*													

^a^ Higher scores indicate an adverse impact on outcomes (i.e., higher depression, anxiety, stress, loneliness). ^b^ Higher scores indicate a positive impact on outcomes (i.e., higher resilience).

**Table 3:** Standardised beta coefficients predicting well-being and resilience (*N* = 1380).

	Depression, Anxiety, and Stress ^a^			Loneliness ^a^			Resilience ^b^		
	*B* (*SE*)	*Beta*	*p*	*B* (*SE*)	*Beta*	*p*	*B* (*SE*)	*Beta*	*p*
Trust in federal government	−0.085 (0.119)	−0.020	0.475	−0.032 (0.017)	−0.058	0.061	0.014 (0.018)	0.021	0.457
Trust in state government	0.190 (0.108)	0.045	0.079	−0.003 (0.016)	−0.006	0.842	0.013 (0.017)	0.021	0.447
Distrust of government in general	0.112 (0.138)	0.021	0.419	0.006 (0.020)	0.010	0.743	−0.057 (0.021)	−0.074	0.008
Interpersonal trust	−1.017 (0.206)	−0.135	<0.001	−0.199 (0.030)	−0.209	<0.001	0.155 (0.032)	0.140	<0.001
COVID-19 beliefs	0.514 (0.117)	0.109	<0.001	−0.021 (0.017)	−0.036	0.207	0.069 (0.018)	0.100	<0.001
IOU: Prospective anxiety	0.242 (0.239)	0.033	0.313	−0.033 (0.034)	−0.035	0.339	0.048 (0.037)	0.045	0.192
IOU: Inhibitory anxiety	1.827 (0.227)	0.311	<0.001	0.191 (0.033)	0.256	<0.001	−0.278 (0.035)	−0.323	<0.001
External locus of control	1.984 (0.269)	0.228	<0.001	0.234 (0.039)	0.212	<0.001	−0.397 (0.042)	−0.310	<0.001
*R* ^2^	0.563			0.429			0.505		

Note: Analyses controlled for gender, age, country of birth, Anglo/non-Anglo background, education, income, subjective social status, employment, living arrangements, remoteness, disability/ies, and whether they were impacted negatively by the 2019–2020 bushfires. ^a^ Higher scores indicate an adverse impact on outcomes (i.e., higher depression, anxiety, stress, loneliness). ^b^ Higher scores indicate a positive impact on outcomes (i.e., higher resilience).

## Data Availability

The data presented in the study are available in Western Sydney University’s Research Direct at https://doi.org/10.26183/v8vd-2v68.

## Supplementary Materials

The following supporting information can be downloaded at: https://www.mdpi.com/article/10.3390/jcm13247495/s1, Table S1: Survey items and sources.

## References

[1] Panchal U., Salazar de Pablo G., Franco M., Moreno C., Parellada M., Arango C., Fusar-Poli P. (2023). The impact of COVID-19 lockdown on child and adolescent mental health: Systematic review. Eur. Child Adolesc. Psychiatry.

[2] Brakefield W.S., Olusanya O.A., White B., Shaban-Nejad A. (2023). Social determinants and indicators of COVID-19 among marginalized communities: A scientific review and call to action for pandemic response and recovery. Disaster Med. Public Health Prep..

[3] Onyeaka H., Anumudu C.K., Al-Sharify Z.T., Egele-Godswill E., Mbaegbu P. (2021). COVID-19 pandemic: A review of the global lockdown and its far-reaching effects. Sci. Prog..

[4] Breslau J., Roth E., Baird M., Carman K., Collins R. (2023). A longitudinal study of predictors of serious psychological distress during COVID-19 pandemic. Psychol. Med..

[5] Tai D., Shah A., Doubeni C., Sia I., Wieland M. (2021). The disproportionate impact of COVID-19 on racial and ethnic minorities in the United States. Clin. Infect. Dis..

[6] Coccia M. (2022). Preparedness of countries to face COVID-19 pandemic crisis: Strategic positioning and factors supporting effective strategies of prevention of pandemic threats. Environ. Res..

[7] Marmot M. (2005). Social determinants of health inequalities. Lancet.

[8] Marmot M. (2015). The health gap: The challenge of an unequal world. Lancet.

[9] Coronavirus (COVID-19) at a Glance—29 November 2020. https://www.health.gov.au/resources/publications/coronavirus-covid-19-at-a-glance-29-november-2020.

[10] Australia’s COVID Response Was One of the Best in the World at First Why Do We Rank so Poorly Now?. https://www.abc.net.au/news/2022-07-28/australia-covid-response-from-good-to-bad/101277358.

[11] Australian Bureau of Statistics Labour Force, Australia. https://www.abs.gov.au.

[12] Australian Bureau of Statistics Trend Unemployment Rate Ends 2019 at 5.1%. https://www.abs.gov.au.

[13] Australian Bureau of Statistics Business Indicators, Business Impacts of COVID-19. https://www.abs.gov.au.

[14] Asmundson G., Taylor S. (2020). Coronaphobia: Fear and the 2019-nCoV outbreak. J. Anxiety Disord..

[15] Courtet P., Olié E., Debien C., Vaiva G. (2020). Keep socially (but not physically) connected and carry on: Preventing suicide in the age of COVID-19. J. Clin. Psychiatry.

[16] Reger M., Stanley I., Joiner T. (2020). Suicide mortality and coronavirus disease 2019—A perfect storm?. JAMA Psychiatry.

[17] Li L., Wang S. (2020). Prevalence and predictors of general psychiatric disorders and loneliness during COVID-19 in the United Kingdom. Psychiatry Res..

[18] Luchetti M., Lee J., Aschwanden D., Sesker A., Strickhouser J., Terracciano A., Sutin A. (2020). The trajectory of loneliness in response to COVID-19. Am. Psychol..

[19] Tull M., Edmonds K., Scamaldo K., Richmond J., Rose J., Gratz K. (2020). Psychological outcomes associated with stay-at-home orders and the perceived impact of COVID-19 on daily life. Psychiatry Res..

[20] Wheaton M., Abramowitz J., Berman N., Fabricant L., Olatunji B. (2012). Psychological predictors of anxiety in response to the H1N1 (swine flu) pandemic. Cogn. Ther. Res..

[21] Hawryluck L., Gold W., Robinson S., Pogorski S., Galea S., Styra R. (2004). SARS control and psychological effects of quarantine, Toronto, Canada. Emerg. Infect. Dis..

[22] Mental Health and Wellbeing during the COVID-19 Period in Australia. https://csrm.cass.anu.edu.au/research/publications/mental-health-and-wellbeing-during-covid-19-period-australia.

[23] Li S., Beames J., Newby J., Maston K., Christensen H., Werner-Seidler A. (2022). The impact of COVID-19 on the lives and mental health of Australian adolescents. Eur. Child Adolesc. Psychiatry.

[24] Devine D., Valgarðsson V., Smith J., Jennings W., Scotto di Vettimo M., Bunting H., McKay L. (2024). Political trust in the first year of the COVID-19 pandemic: A meta-analysis of 67 studies. J. Eur. Public Policy.

[25] Douglas K., Uscinski J., Sutton R., Cichocka A., Nefes T., Ang C., Deravi F. (2019). Understanding conspiracy theories. Political Psychol..

[26] Southwell B., Niederdeppe J., Cappella J., Gaysynsky A., Kelley D., Oh A., Peterson E., Chou W. (2019). Misinformation as a misunderstood challenge to public health. Am. J. Prev. Med..

[27] Kużelewska E., Tomaszuk M. (2022). Rise of conspiracy theories in the pandemic times. Int. J. Semiot. Law-Rev. Int. Sémiotique Jurid..

[28] Pickles K., Cvejic E., Nickel B., Copp T., Bonner C., Leask J., Ayre J., Batcup C., Cornell S., Dakin T. (2021). COVID-19 misinformation trends in Australia: Prospective longitudinal national survey. J. Med. Internet Res..

[29] Pummerer L., Böhm R., Lilleholt L., Winter K., Zettler I., Sassenberg K. (2022). Conspiracy theories and their societal effects during the COVID-19 pandemic. Soc. Psychol. Personal. Sci..

[30] Hughes J.P., Efstratiou A., Komer S.R., Baxter L.A., Vasiljevic M., Leite A.C. (2022). The impact of risk perceptions and belief in conspiracy theories on COVID-19 pandemic-related behaviours. PLoS ONE.

[31] Bavolar J., Kacmar P., Hricova M., Schrötter J., Kovacova-Holevova B., Köverova M., Raczova B. (2023). Intolerance of uncertainty and reactions to the COVID-19 pandemic. J. Gen. Psychol..

[32] Haywood D., Mason O. (2023). Perception of COVID-19 threat, low self-efficacy, and external locus of control lead to psychological distress during the COVID-19 pandemic. Psychol. Health Med..

[33] Denson N., Dunn K., Ben J., Kamp A., Sharples R., Pitman D., Paradies Y., McGarty C. Australians’ well-being and resilience during COVID-19. https://www.crisconsortium.org/research-reports/covid-resilience.

[34] Kelly A., Halford W. (2007). Responses to ethical challenges in conducting research with Australian adolescents. Aust. J. Psychol..

[35] Bruzzese J.M., Fisher C.B. (2003). Assessing and enhancing the research consent capacity of children and youth. Appl. Dev. Sci..

[36] Australian Bureau of Statistics (2021). Population: Census—Information on Sex and Age. https://www.abs.gov.au.

[37] Australian Bureau of Statistics (2021). Income and Work: Census—Information on Income, Occupation and Employment. https://www.abs.gov.au.

[38] (2021). Australia’s Population by Country of Birth—Statistics on Australia’s Estimated Resident Population by Country of Birth. https://www.abs.gov.au.

[39] Australian Bureau of Statistics Education and Training: Census, 2021—Information on Qualifications, Educational Attendance and Type of Educational Institution. https://www.abs.gov.au.

[40] Australian Bureau of Statistics Historical Population, 2021—Demographic Data Going Back as Far as Data is Available. https://www.abs.gov.au.

[41] Kyriazos T., Stalikas A., Prassa K., Yotsidi V. (2018). Can the Depression Anxiety Stress Scales Short be shorter? Factor structure and measurement invariance of DASS-21 and DASS-9 in a Greek, non-clinical sample. Psychology.

[42] Hays R., DiMatteo M. (1987). A short-form measure of loneliness. J. Personal. Assess..

[43] Smith B., Dalen J., Wiggins K., Tooley E., Christopher P., Bernard J. (2008). The brief resilience scale: Assessing the ability to bounce back. Int. J. Behav. Med..

[44] Miller A. (1974). Political issues and trust in government: 1964–1970. Am. Political Sci. Rev..

[45] Goldberg L., Johnson J., Eber H., Hogan R., Ashton M., Cloninger C., Gough H. (2006). The international personality item pool and the future of public-domain personality measures. J. Res. Personal..

[46] Carleton R., Norton M., Asmundson G. (2007). Fearing the unknown: A short version of the Intolerance of Uncertainty Scale. J. Anxiety Disord..

[47] Craig A., Franklin J., Andrews G. (1984). A scale to measure locus of control of behaviour. Br. J. Med. Psychol..

[48] Reaching for a Healthier Life: Facts on Socioeconomic Status and Health in the US. http://www.macses.ucsf.edu/news/Reaching%20for%20a%20Healthier%20life.pdf.

[49] Royal Commission into National Natural Disaster Arrangements Report. https://www.royalcommission.gov.au/natural-disasters/report.

[50] Hardship, Distress, and Resilience: The Initial Impacts of COVID-19 in Australia. https://csrm.cass.anu.edu.au/research/publications/hardship-distress-and-resilience-initial-impacts-covid-19-australia-1.

[51] Fisher J., Tran T., Hammarberg K., Sastry J., Nguyen H., Rowe H., Popplestone S., Stocker R., Stubber C., Kirkman M. (2020). Mental health of people in Australia in the first month of COVID-19 restrictions: A national survey. Med. J. Aust..

[52] Rossell S., Neill E., Phillipou A., Tan E., Toh W., Van Rheenen T., Meyer D. (2021). An overview of current mental health in the general population of Australia during the COVID-19 pandemic: Results from the COLLATE project. Psychiatry Res..

[53] Zhao Y., Leach L., Walsh E., Batterham P., Calear A., Phillips C., Olsen A., Doan T., LaBond C., Banwell C. (2022). COVID-19 and mental health in Australia—A scoping review. BMC Public Health.

[54] Shiloh S., Peleg S., Nudelman G. (2022). Core self-evaluations as resilience and risk factors of psychological distress during the COVID-19 pandemic. Psychol. Health Med..

[55] Shoychet G., Lenton-Brym A., Antony M. (2022). The impact of COVID-19 anxiety on quality of life in Canadian adults: The moderating role of intolerance of uncertainty and health locus of control. Can. J. Behav. Sci. Rev. Can. Sci. Comport..

[56] Gullo S., Gelo O., Bassi G., Lo Coco G., Lagetto G., Esposito G., Pazzagli C., Salcuni S., Mazzeschi C., Giordano C. (2023). The role of emotion regulation and intolerance to uncertainty on the relationship between fear of COVID-19 and distress. Curr. Psychol..

[57] Sigurvinsdottir R., Thorisdottir I., Gylfason H. (2020). The impact of COVID-19 on mental health: The role of locus on control and internet use. Int. J. Environ. Res. Public Health.

[58] Krampe H., Danbolt L., Haver A., Stålsett G., Schnell T. (2021). Locus of control moderates the association of COVID-19 stress and general mental distress: Results of a Norwegian and a German-speaking cross-sectional survey. BMC Psychiatry.

[59] Yuan H., Long Q., Huang G., Huang L., Luo S. (2022). Different roles of interpersonal trust and institutional trust in COVID-19 pandemic control. Soc. Sci. Med..

[60] Choi K., Jung J., Kim H. (2023). Political trust, mental health, and the coronavirus pandemic: A cross-national study. Res. Aging.

[61] Putnam R. (2000). Bowling Alone: The Collapse and Revival of American Community.

[62] Hudson J. (2006). Institutional trust and subjective well-being across the EU. Kyklos Int. Rev. Soc. Sci..

[63] Kamp A., Dunn K., Sharples R., Denson N., Diallo T. (2023). Understanding Trust in Contemporary Australia Using Latent Class Analysis. Cosmop. Civ. Soc. Interdiscip. J..

[64] Putnam R. (1993). What makes democracy work?. Natl. Civ. Rev..

[65] Fukuyama F. (1996). Trust: The Social Virtues and the Creation of Prosperity.

[66] Moesen W., Van Puyenbroeck T., Cherchye L. (2000). Trust as Societal Capital: Economic Growth in European Regions.

[67] Zak P., Knack S. (2001). Trust and growth. Econ. J..

[68] Fazio G., Giambona F., Vassallo E., Vassiliadis E. (2018). A measure of trust: The Italian regional divide in a latent class approach. Soc. Indic. Res..

[69] 2020 Edelman Trust Barometer. https://www.edelman.com/trust/2020-trust-barometer.

[70] 2021 Edelman Trust Barometer. https://www.edelman.com/trust/2021-trust-barometer.

[71] 2022 Edelman Trust Barometer. https://www.edelman.com.au/sites/g/files/aatuss381/files/2022-02/Edelman%20Trust%20Barometer%202022%20-%20Australia%20Country%20Report.pdf.

[72] Douglas K. (2021). COVID-19 conspiracy theories. Group Process. Intergroup Relat..

[73] Douglas K., Sutton R., Cichocka A. (2017). The psychology of conspiracy theories. Curr. Dir. Psychol. Sci..

[74] Faulkner J., O’brien W., Stuart B., Stoner L., Batten J., Wadsworth D., Askew C., Badenhorst C., Byrd E., Draper N. (2022). Physical activity, mental health and wellbeing of adults within and during the easing of COVID-19 restrictions, in the United Kingdom and New Zealand. Int. J. Environ. Res. Public Health.

[75] Allport G. (1954). The Nature of Prejudice.

[76] Bach R., Kaufman D., Settle K., Duckworth M. (2015). Policy leadership challenges in supporting community resilience. Strategies for Supporting Community Resilience: Multinational Experiences: CRISMART 2015.

[77] Georgiou N., Delfabbro P., Balzan R. (2020). COVID-19-related conspiracy beliefs and their relationship with perceived stress and pre-existing conspiracy beliefs. Personal. Individ. Differ..

[78] Miller J., Saunders K., Farhart C. (2016). Conspiracy endorsement as motivated reasoning: The moderating roles of political knowledge and trust. Am. J. Political Sci..

[79] Van Prooijen J., Douglas K. (2018). Belief in conspiracy theories: Basic principles of an emerging research domain. Eur. J. Soc. Psychol..

[80] Agley J., Xiao Y. (2021). Misinformation about COVID-19: Evidence for differential latent profiles and a strong association with trust in science. BMC Public Health.

[81] Peucker M., Fisher T. (2023). Mainstream media use for far-right mobilisation on the alt-tech online platform Gab. Media Cult. Soc..

[82] Fawzi N. (2019). Untrustworthy news and the media as “enemy of the people”? How a populist worldview shapes recipients’ attitudes toward the media. Int. J. Press Politics.

[83] Haller A., Holt K. (2019). Paradoxical populism: How PEGIDA relates to mainstream and alternative media. Inform. Commun. Soc..

[84] Stecula D., Pickup M. (2021). How populism and conservative media fuel conspiracy beliefs about COVID-19 and what it means for COVID-19 behaviors. Res. Politics.

[85] Peucker M., Spaaij R. (2023). Alternative epistemology in far-right anti-publics: A qualitative study of Australian activists. Int. J. Politics Cult. Soc..

[86] Maguire P., Looi J. (2022). Moral injury and psychiatrists in public community mental health services. Australas. Psychiatry.

[87] Biddlestone M., Green R., Douglas K. (2020). Cultural orientation, power, belief in conspiracy theories, and intentions to reduce the spread of COVID-19. Br. J. Soc. Psychol..

[88] The Guardian Words Matter: How New Zealand’s Clear Messaging Helped Beat Covid. https://www.theguardian.com/world/2021/feb/26/words-matter-how-new-zealands-clear-messaging-helped-beat-covid.

[89] Covid Australia: Sydney Celebrates End of 107-Day Lockdown. https://www.bbc.com/news/world-australia-58866464.

[90] Melbourne Marks 200 Days of COVID-19 Lockdowns Since the Pandemic Began—ABC News. https://www.abc.net.au/news/2021-08-19/melbourne-200-days-of-covid-lockdowns-victoria/100386078.

[91] WA Has a ‘Go Hard, Go Early’ Approach to COVID-19 Lockdowns, But What Is the Pathway Out?. https://www.abc.net.au/news/2021-07-18/wa-hard-lockdown-approach-pathway-out-of-covid/100301206.

[92] Liu C., Zhang E., Wong G., Hyun S. (2020). Factors associated with depression, anxiety, and PTSD symptomatology during the COVID-19 pandemic: Clinical implications for US young adult mental health. Psychiatry Res..

[93] Mpox Is on the Rise in Australia, Prompting Calls to Limit Intimate Partners and Keep Contact Tracing Details—ABC News. https://www.abc.net.au/news/2024-07-26/mpox-cases-outbreak-australia-victoria-spike-infections/104119360.

