# Supplementary material for: Australians’ Well-Being and Resilience During COVID-19: The Role of Trust, Misinformation, Intolerance of Uncertainty, and Locus of Control
Source: J Clin Med. 2024 Dec 10;13(24):7495. doi: 10.3390/jcm13247495 (PMC11677133; doi:10.3390/jcm13247495)
Supplement: Supplementary file 1 [file jcm-13-07495-s001.zip › jcm-3235276-supplementary.pdf]

Supplementary Table S1. Survey items and sources

Survey Item/Questions	Source
**Depression, Anxiety & Stress** (from 0 = never to 3 = almost always)<br><br>**Depression**<br>- I found it difficult to work up the initiative to do things<br>- I felt that I had nothing to look forward to<br>- I was unable to become enthusiastic about anything<br><br>**Anxiety**<br>- I experienced trembling (e.g., in the hands)<br>- I was worried about situations in which I might panic and make a fool of myself<br>- I felt I was close to panic<br><br>**Stress**<br>- I tended to over-react to situations<br>- I found myself getting agitated<br>- I was intolerant of anything that kept me from getting on with what I was doing	Depression, Anxiety and Stress Scale (DASS-9) (Kyriazos et al., 2018)
**Loneliness** (from 1 = never to 4 = often)<br><br>I lack companionship<br><br>There is no one I can turn to<br><br>I am an outgoing person (reverse)<br><br>I feel left out<br><br>I feel isolated from others<br><br>I can find companionship when I want it (reverse)<br><br>I am unhappy being so withdrawn<br><br>People are around me but not with me	UCLA Loneliness Scale (ULS-8) (Hays & DiMatteo, 1987)
**Resilience** (from 1 = strongly disagree to 5 = strongly agree)<br><br>I tend to bounce back quickly after hard times<br><br>I have a hard time making it through stressful events (reverse)<br><br>It does not take me long to recover from a stressful event<br><br>It is hard for me to snap back when something bad happens (reverse)<br><br>I usually come through difficult times with little trouble<br><br>I tend to take a long time to get over set-backs in my life (reverse)	Brief Resilience Scale (Smith, 2008)

**Trust in federal government** (from 1 = strongly disagree to 7 = strongly agree)<br><br>Scott Morrison is doing a great job during the COVID-19 crisis<br><br>Federal Parliament is doing a great job during the COVID-19 crisis<br><br>The federal government is providing me with sufficient information about who should be tested for COVID-19<br><br>The federal government is providing me with sufficient information about where to be tested for COVID-19<br><br>The federal government is providing me with sufficient information about who should be isolated for COVID-19	Original
**Trust in state government** (from 1 = strongly disagree to 7 = strongly agree)<br><br>My State Parliament is doing a great job during the COVID-19 crisis<br><br>My State government is providing me with sufficient information about who should be tested for COVID-19<br><br>My State government is providing me with sufficient information about where to be tested for COVID-19<br><br>My State government is providing me with sufficient information about who should be isolated for COVID-19	Original
**Distrust of government in general** (from 1 = strongly disagree to 7 = strongly agree)<br><br>The government does what is right (reverse)<br><br>The government is regulated by a few big interests<br><br>The government wastes considerable tax money<br><br>Most government officials are not capable of performing their duties<br><br>Many government officials are dishonest	Adapted from the Political Cynicism Scale (Miller, 1974)
**Interpersonal trust** (from 1 = very inaccurate to 5 = very accurate)<br><br>Trust what people say<br><br>Trust others<br><br>Believe in human goodness<br><br>Distrust people (reverse)<br><br>Suspect hidden motives in others (reverse)<br><br>Believe that people are essentially evil (reverse)	Trusting Scale from the International Personality Item Pool (https://ipip.ori.org/) (Goldberg et al., 2006)

**COVID-19 beliefs (including conspiracy theories and misinformation)** (from 1 = strongly disagree to 7 = strongly agree)<br><br>COVID-19 was created by the Chinese as a weapon to be used against Western countries<br><br>COVID-19 is a bio-engineered virus<br><br>Hot climates/temperatures kill the coronavirus<br><br>There is strong evidence that ibuprofen and other anti-inflammatory drugs accelerate the multiplication of the virus<br><br>Home remedies (e.g., essential oils, gargling with vinegar and water, inhaling hot air, and some vitamins and teas) can kill the virus<br><br>Face masks do not prevent the spread of COVID-19<br><br>Making faces masks mandatory impedes my personal freedom<br><br>COVID-19 is worsened through installing 5G towers<br><br>The risk of COVID-19 infection is lower than what the government says<br><br>The number of deaths due to COVID-19 is overstated	Original
**Intolerance of Uncertainty (IOU)** (from 1 = not at all characteristic of me to 5 = entirely characteristic of me)<br><br>**Inhibitory anxiety**<br><br>- Uncertainty keeps me from living a full life<br>- When it's time to act, uncertainty paralyses me<br>- When I am uncertain I can't function very well<br>- The smallest doubt can stop me from acting<br>- I must get away from all uncertain situations<br><br>**Prospective anxiety**<br><br>- Unforeseen events upset me greatly<br>- It frustrates me not having all the information I need<br>- One should always look ahead so as to avoid surprises<br>- A small unforeseen event can spoil everything, even with the best of planning<br>- I always want to know what the future has in store for me<br>- I can't stand being taken by surprise<br>- I should be able to organize everything in advance	Intolerance of Uncertainty Scale (Carleton et al., 2007)
**External locus of control (LOC)** (from 1 = strongly disagree to 6 = strongly agree)<br><br>I can anticipate difficulties and take action to avoid them (reverse)<br><br>A great deal of what happens to me is probably just a matter of chance<br><br>Everyone knows that luck or chance determines one's future<br><br>I can control my problem(s) only if I have outside support	Locus of control of behaviour (Craig et al., 1984)

When I make plans, I am almost certain that I can make them work (reverse)<br><br>My problem(s) will dominate me all my life<br><br>My mistake and problems are my responsibility to deal with (reverse)<br><br>Becoming a success is a matter of hard work, luck has little or nothing to do with it (reverse)<br><br>My life is controlled by outside actions and events<br><br>People are victims of circumstances beyond their control<br><br>To continually manage my problems I need professional help<br><br>When I am under stress, the tightness in my muscles is due to things outside my control<br><br>I believe a person can really be the master of his fate (reverse)<br><br>It is impossible to control my irregular and fast breathing when I am having difficulties<br><br>I understand why my problem(s) varies so much from one occasion to the next<br><br>I am confident of being able to deal successfully with future problems (reverse)<br><br>In my case maintaining control over my problem(s) is due mostly to luck	
**Gender**<br>- Female<br>- Male<br>- Non-binary<br>- Other<br>- Prefer not to say	Original
**Age**<br>- 16-17<br>- 18-24<br>- 25-34<br>- 35-44<br>- 45-54<br>- 55-64<br>- 65-74<br>- 75 or older	Original
**Country of birth**<br>- Australia<br>- Other	Original
**Do you identify with an Anglo (e.g., White Australian/British) background?**<br>- Yes<br>- No	Original
**Highest level of education completed**	Original

- No formal qualifications<br>- Higher School Certificate (year 12) or equivalent<br>- Trade or TAFE qualification<br>- Other tertiary qualification<br>- University degree<br>- Postgraduate qualification<br>- Other	
**What is your personal income, before tax?**<br>- Under $20,000<br>- $20,000 - $29,999<br>- $30,000 - $39,999<br>- $40,000 - $49,999<br>- $50,000 - $59,999<br>- $60,000 - $79,999<br>- $80,000 - $99,999<br>- $100,000 - $149,999<br>- $150,000 or more<br>- Don’t know / Don’t wish to divulge	Original
**Subjective Social Status**<br><br><u>Instructions</u>: Think of this ladder as representing where people stand in Australia. At the top of the ladder are the people who are the best off – those who have the most money, the most education, and the most respected jobs. At the bottom are the people who are the worst off – those who have the least money, least education, the least respected jobs, or no job. The higher up you are on this ladder, the closer you are to the people at the very top; the lower you are, the closer you are to the people at the very bottom.<br><br>Please indicate the rung where you think you stand at this time in your life relative to other people in Australia (from 1 = ‘bottom’ to 10 = ‘top’).<br><br>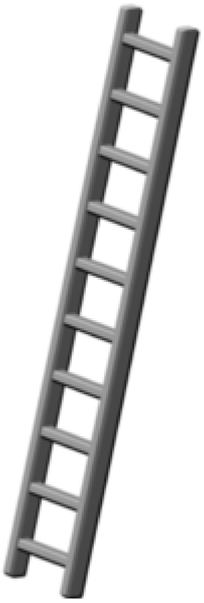	Adapted from the MacArthur Scale of Subjective Social Status (Adler & Stewart, 2007)

**Employment status**<br>- Employed<br>- Unemployed<br>- Self-employed<br>- Retired<br>- Caring/home duties<br>- Student<br>- Other	Original
**Which of the following best describes your living situation**<br>- Single person, living alone<br>- Single person, living with parents/family<br>- Single person, living with one or more children<br>- Couple living with one or more children<br>- Couple living without children<br>- Share house (group home of unrelated adults)<br>- Other	Original
**Remoteness**<br>- Major city<br>- Inner regional<br>- Outer regional<br>- Remote<br>- Very remote	Original
**Do you have a disability/ies?**<br>- Yes<br>- No	Original
**Please indicate on the following scale how negatively impacted you were by the Australian 2019-20 bushfires** (from 1 = not at all to 4 = extremely so)<br><br>Housing<br><br>Physical health<br><br>Mental health<br><br>Emotional health<br><br>Health of someone close to you	Original

References

Adler, N.E., & Stewart, J. (2007). *The MacArthur Scale of Subjective Social Status.* MacArthur SES Health Netw: San Francisco, CA, USA. https://macses.ucsf.edu/research/psychosocial/subjective.php

Carleton, R. N., Norton, M. P. J., & Asmundson, G. J. (2007). Fearing the unknown: A short version of the Intolerance of Uncertainty Scale. *Journal of anxiety disorders*, *21*(1), 105-117.

Craig, A. R., Franklin, J. A., & Andrews, G. (1984). A scale to measure locus of control of behaviour. *British Journal of Medical Psychology*, *57*(2), 173-180.

Goldberg, L. R., Johnson, J. A., Eber, H. W., Hogan, R., Ashton, M. C., Cloninger, C. R., & Gough, H. G. (2006). The international personality item pool and the future of public-domain personality measures. *Journal of Research in personality*, *40*(1), 84-96.

Hays, R. D., & DiMatteo, M. R. (1987). A short-form measure of loneliness. *Journal of personality assessment*, *51*(1), 69-81.

Kyriazos, T. A., Stalikas, A., Prassa, K., & Yotsidi, V. (2018). Can the Depression Anxiety Stress Scales Short be shorter? Factor structure and measurement invariance of DASS-21 and DASS-9 in a Greek, non-clinical sample. *Psychology*, *9*(5), 1095-1127.

Miller, A. H. (1974). Political issues and trust in government: 1964–1970. *American political science review*, *68*(3), 951-972.

Smith, B. W., Dalen, J., Wiggins, K., Tooley, E., Christopher, P., & Bernard, J. (2008). The brief resilience scale: assessing the ability to bounce back. *International journal of behavioral medicine*, *15*(3), 194-200.
